# Genome-wide identification of *GH28* family and insight into its contributions to pod shattering resistance in *Brassica napus* L.

**DOI:** 10.1186/s12864-024-10406-y

**Published:** 2024-05-17

**Authors:** Fugui Zhang, Nian Liu, Tianhua Chen, Hong Xu, Rui Li, Liyan Wang, Shuo Zhou, Qing’ao Cai, Xinzhe Hou, Ling Wang, Xingzhi Qian, Zonghe Zhu, Kejin Zhou

**Affiliations:** College of Agronomy, Anhui Agricultural University, 130, Changjiang West Road, Hefei, Anhui 230036 China

**Keywords:** Rapeseed, Glycosyl hydrolase family 28 (GH28), Pod shattering resistance, Polygalacturonase

## Abstract

Rapeseed (*Brassica napus* L.), accounts for nearly 16% of vegetable oil, is the world’s second produced oilseed. However, pod shattering has caused significant yield loses in rapeseed production, particularly during mechanical harvesting. The *GH28* genes can promote pod shattering by changing the structure of the pod cell wall in *Arabidopsis*. However, the role of the *GH28* gene family in rapeseed was largely unknown. Therefore, a genome-wide comprehensive analysis was conducted to classify the role of *GH28* gene family on rapeseed pod shattering. A total of 37 *BnaGH28* genes in the rapeseed genome were identified. These *BnaGH28s* can be divided into five groups (Group A-E), based on phylogenetic and synteny analysis. Protein property, gene structure, conserved motif, cis-acting element, and gene expression profile of *BnaGH28* genes in the same group were similar. Specially, the expression level of genes in group A-D was gradually decreased, but increased in group E with the development of silique. Among eleven higher expressed genes in group E, two *BnaGH28* genes (*BnaA07T0199500ZS* and *BnaC06T0206500ZS*) were significantly regulated by IAA or GA treatment. And the significant effects of *BnaA07T0199500ZS* variation on pod shattering resistance were also demonstrated in present study. These results could open a new window for insight into the role of *BnaGH28* genes on pod shattering resistance in rapeseed.

**Supplementary Information:**

The online version contains supplementary material available at 10.1186/s12864-024-10406-y.

## Introduction

Rapeseed (*Brassica napus L*., AACC, 2n = 38) is the second largest oil crop, accounting for about 16% of the total global vegetable oil production in the world [1]. Rapeseed pods readily dehisce and disperse their seeds at maturity, resulting in yield losses of 15–50% under unfavorable weather or machine harvesting conditions [2]. Therefore, cultivating new cultivars with pod shattering resistance is the most effective approach to reducing the yield losses in rapeseed production [3].

During pod shattering, cell separation in the dehiscence zone (DZ) is thought to caused by pectin degradation [4]. Polygalacturonase (PG), a member of the Glycoside Hydrolase family 28 (GH28), can catalyze the hydrolysis of α-(1–4) galactosidic bond cleavage and D-glucuronic acid bond in pectin molecules, resulting in cell separation [5, 6]. Especially, *RABIDOPSIS DEHISCENCE ZONE POLYGALACTURONASE 1* (*ADPG1*) and *ADPG2* were involved in the cell separation during *Arabidopsis* silique dehiscence [7]. The role of PG in pod shattering was also been reported in pea and soybean [8, 9]. Besides, *QUARTET2* (*QRT2*), another member of the *GH28* family, was involved in anther dehiscence and floral organ abscission in *Arabidopsis* [10]. *QRT3* with polygalacturonase activity plays a direct role in the degradation of pollen mother cell wall [11].

In *Brassica* plant*s*, a large number of studies have been carried out to investigate the mechanism of pod shattering (resistance). So far, eight pod shattering resistance related genes, including *SHATTERPROOF1/2* (*SHP1/2*) [12], *FRUITFULL* (*FUL*) [13], *INDEHISCENT* (*IND*) [14], *ALCATRAZ* (*ALC*) [15], *NAC SECONDARY WALL THICKENING PROMOTING FACTOR 1/2* (*NST1/2*) [16], *POLYGlACTOURANAZE* (*PG*) [17], *REPLUMLESS* (*RPL*) [18] and *SPATULA *(*SPT)* [19] have been reported. However, the effects of pectin and PG on rapeseed pod shattering were largely unknown.

Thus, investigating the role of *GH28* on pod shattering in *B. napus* is of great significance in understanding the pod shattering mechanism. In present study, 37 *BnaGH28* genes were identified in the *B. napus* genome by a homology sequence blast. The protein physicochemical properties, conserved motif, gene structure, cis-acting element, and tissue expression profile analysis were also conducted. Furthermore, Two *BnaGH28* genes (*BnaA07T0199500ZS* and *BnaC06T0206500ZS*) significantly regulated by IAA or GA treatment were be found in present study. And the significant effects of *BnaA07T0199500ZS* variation on pod shattering resistance were also demonstrated. These findings could provide key information for developing pod shattering resistant cultivars by genetic approach in *B. napus.*

## Materials and methods

### Identification of *BnaGH28* genes in the *B. napus* genome

For the Identification of *BnaGH28* genes in the *B. napus* genome, a blastP analysis was performed in the *Brassica napus* pan-genome Information Resource (BnPIR) database [20] (http://cbi.hzau.edu.cn/bnapus/index.php) by using ten AtGH28 protein reference sequences from UniProt database [21](https://www.uniprot.org/). And then, BnaGH28s were screened according to the conserved protein structure by using the Simple Modular Architecture Research Tool (SMART) [22] (http://smart.embl.de/smart/set_mode.cgi?NORMAL=1/). For chromosome location analysis, the *B. napus* genome file, chromosome annotation information, and location information of *BnaGH28* genes were downloaded from the BnPIR database. The Gene Location Visualization subroutine of the TBtools software was used to map the chromosome location of *BnaGH28* genes.

### Evolutionary relationship of the GH28 family

The full-length protein sequences of 10 AtGH28s, and 37 BnaGH28s were obtained from the UniProt database and BnPIR database, respectively. Protein sequences were aligned using the ClustalW program. The neighbor-joining (NJ) phylogenetic tree was constructed by the MEGA software. Bootstrap analysis was conducted with 1000 replications. Then the evolutionary tree was visualized by the online website iTOL [23] ( https://itol.embl.de/ ).

### Analysis of collinearity replication relationship of *BnaGH28* genes

To analyze the replication events involved in *BnaGH28* genes between or within species, the genome and annotation files of *A. thaliana* and *B. napus* were obtained from the Ensemble Plants database (https://plants.ensembl.org/index.html) and BnPIR database, respectively. The OneStepMCScanX program of TBtools was used to analyze the collinearity between or within species of *BnaGH28* genes.

### Physicochemical properties of BnaGH28 proteins

The physicochemical properties including the number of amino acids (AA), molecular weight (MW), isoelectric point (pI), instability index (II), and grand average of hydropathicity (GRAVY) of BnaGH28 proteins were analyzed by the online software Expasy [24] (https://web.expasy.org/protparam/). Subcellular localization (SL) of BnaGH28 proteins was predicted by the online software Wolf PSORT [25] ( https://wolfpsort.hgc.jp/ ).

### Conserved motifs and gene structure of the GH28 family

The online analysis software MEME [26] ( https://meme-suite.org/meme/ ) was used to analyze the conserved motifs of all BnaGH28 protein sequences, with the following parameters: the optimum width of motif, 6–50; the number of repetitions, any; the number of motifs, 20; the number of motif occurrences on each sequence is not limited. Then visualization was conducted by the TBtools software.

The rapeseed genome and annotation files were downloaded from the BnPIR database. The *BnaGH28* gene structure was visualized by the subroutine Gene Structure View function of TBtools software.

### Tissue expression profiles of *BnaGH28* genes

In order to investigate the tissue expression profiles of *BnaGH28* genes, the gene expression data in the root, stem, leaf, bud, filament, petal, pollen, sepal, cotyledon, seed, and silique of *B. napus* was obtained from the BnTIR database ( http://yanglab.hzau.edu.cn ).

### Cis-acting elements and gene expression respond to phytohormone

For cis-acting elements analysis, the upstream 2000 bp promoter sequence of *BnaGH28* genes was extracted from the *B. napus* genome by the TBtools software. Cis-acting elements in *BnaGH28* promoter regions were predicted by the online software Plant CARE [27] (http://bioinformatics.psb.ugent.be/webtools/plantcare/html/).

Primers were designed using qPrimerDB, and the specific primer sequence is shown in Table S1. In our preliminary experiment, 0–45 mg/L gibberellic acid 3 (GA3) or 0-500 mg/L auxin (IAA) were sprayed in rapeseed. Both 15 mg/L GA3 and 500 mg/L IAA could significantly increase pod shattering resistance in rapeseed (Fig. S1). For investigating *BnaGH28* gene expression response to phytohormone, 15 mg/L GA3 or 500 mg/L IAA was sprayed in the silique fifteen days after flowering. Pods were collected for gene expression analysis after 7 days.

### Variation analysis of *BnaGH28*

To analyze the effects of *BnaGH28* gene variation on pod shattering resistance, the variation of two candidate genes and pod shattering resistance were investigated in a rapeseed micro-core collection. The sequence variation and haplotype information were extracted from our previous study (Table S2) [28]. During an evaluation of pod shattering resistance, a stem strength tester (YYD-1 A, Zhejiang Top Cloud-agri Technology Co., Ltd) was employed in the present study. Ten uniform fresh harvest siliques per accession were used to evaluate pod shattering resistance. The maximum pressure value of the middle 3 cm interval was recorded as the pod shattering force.

## Results

### Identification of *BnaGH28* genes in the *B. napus* genome

**Fig. 1:**
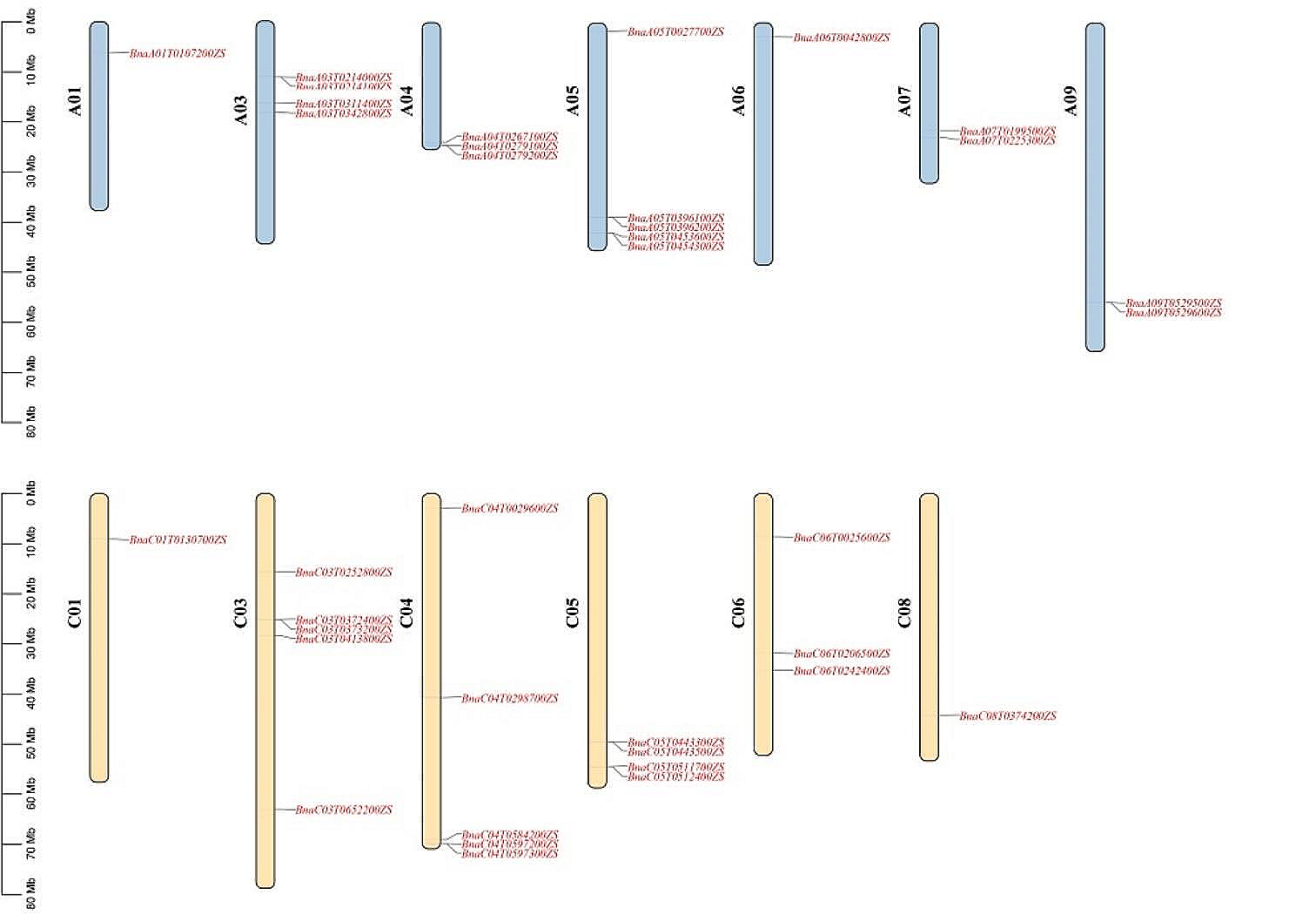
Distribution of *BnaGH28* genes on *Brassica napus* chromosomes

To identify the GH28 family members in *B. napus*, ten AtGH28 protein sequences were used as query sequences for BLASTP analysis. A total of 37 *BnaGH28* genes were identified in the *B. napus* genome based on transmembrane structure and conversed domain structures (Table S3). Chromosomal location results have shown that 37 *BnaGH28* genes were unevenly distributed on 13 chromosomes. There were five *BnaGH28* genes located on the chromosome A05, C03, and C04. There was only one *BnaGH28* on chromosomes A01, A06, C01, and C08 (Fig. 1). It was interesting that the distribution of *BnaGH28* genes on the A or C sub-genome of *B. napus* was similar. There were 18 and 19 *GH28* genes located in the A and C sub-genome, respectively.

### Evolutionary relationship of GH28 family

**Fig. 2:**
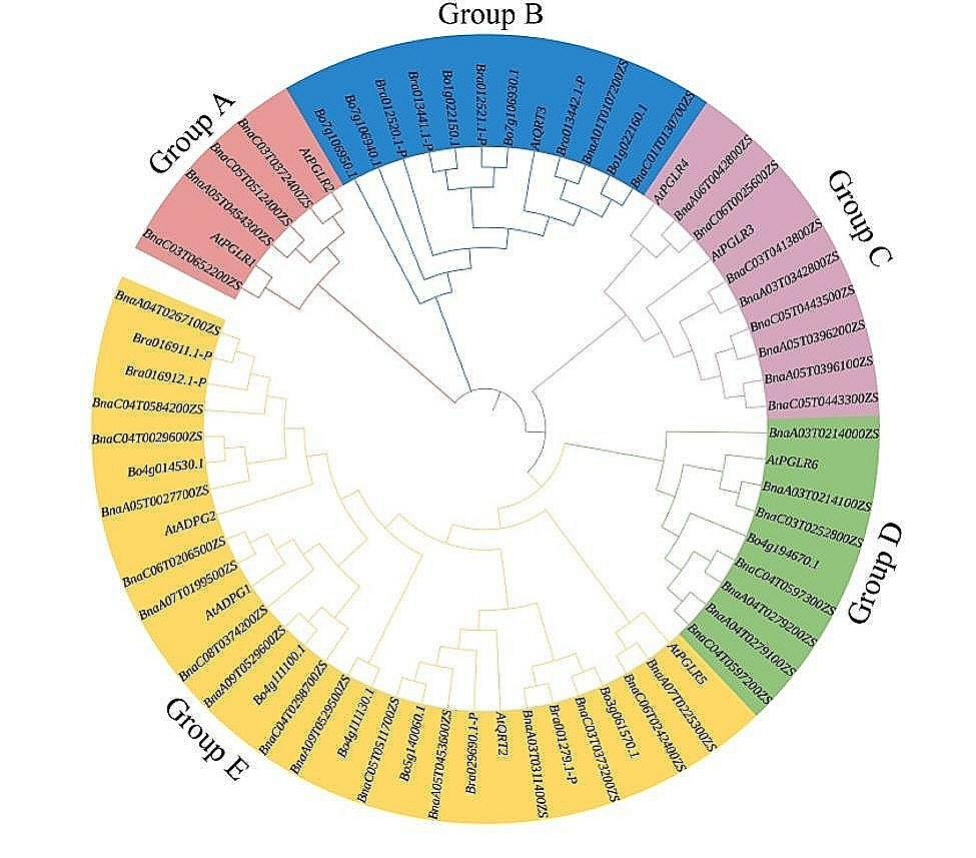
Phylogenetic tree of BnaGH28, BraGH28, BolGH28 and AtGH28 proteins

To determine the evolutionary relationships of the GH28 family, an unrooted phylogenetic tree was constructed between 66 GH28s (37 from *B. napus*, 10 from *A. thaliana*, 8 from *B. rapa*, and 11 from *B. oleracea*) by MEGA software with NJ method. Results indicated that the 66 GH28s were grouped into five groups (Group A, B, C, D, and E) (Fig. 2). Group A contained 6 GH28 members (2 AtGH28s, 4 BnaGH28s), Group B contained 12 GH28 members (1 AtGH28, 2 BnaGH28s, 4 BraGH28s, 5 BolGH28s), Group C contained 10 GH28 members (2 AtGH28s, 8 BnaGH28s), Group D contained 9 GH28 members (1 AtGH28, 7 BnaGH28s, 1 BolGH28), and Group E contained 29 GH28 members (4 AtGH28s, 16 BnaGH28s, 4 BraGH28s, 5 BolGH28s).

**Fig. 3:**
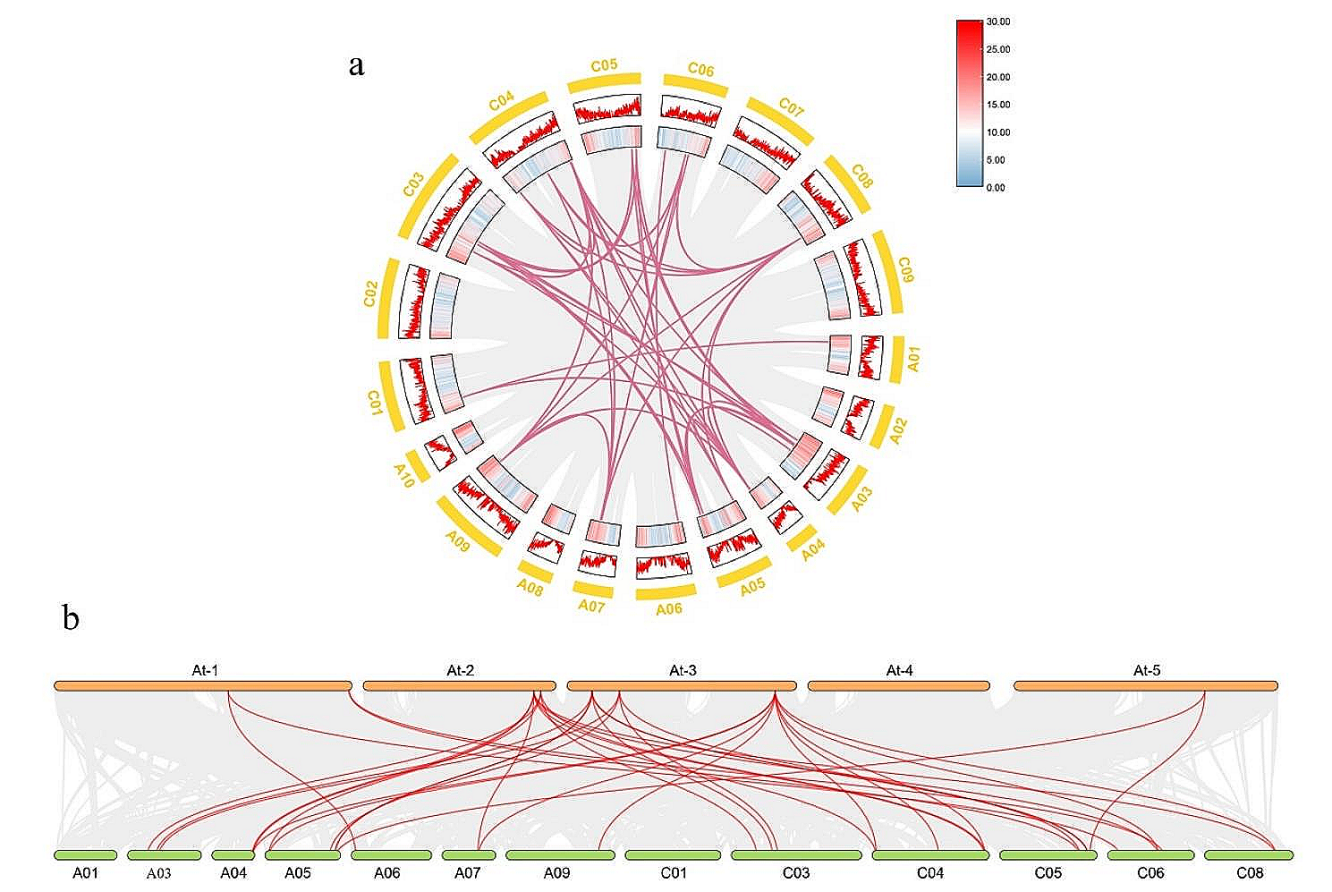
Synteny analysis of *GH28* among *Arabidopsis thaliana* and *Brassica napus* | (**a**) Intraspecies collinearity analysis; (**b**) Interspecies collinearity analysis. The coarse red line in the circle is the collinearity gene of *GH28* genes among species, and the fine gray line indicates all the collinearity genes contained among species

In order to study the replication events of the *BnaGH28* gene family in the *B. napus* genome, the interspecies collinearity of *A. thaliana* and *B. napus* and intraspecies collinearity were investigated by the TBtools software (Fig. 3). A large number of orthologous *GH28* genes between *B. napus* and *A. thaliana* were identified in the present study (Fig. 3b). Within *B. napus* genome, the *GH28* gene has been amplified to a certain extent (Fig. 3a). These indicating that *GH28* gene replications might play a very important role in the development of *B. napus*.

### Physicochemical properties of BnaGH28 proteins

In order to clarify the basic characteristics of BnaGH28 proteins, the AA, MW, pI, II, GRAVY, and SL of 37 BnaGH28 proteins were analyzed (Table S4). The length of BnaGH28 proteins ranged from 144 to 546 AA, with an average length of 411 AA. Their MW was ranged from 15501.33 (BnaA09T0529500ZS) to 58893.73 Da (BnaC06T0206500ZS). The pI was ranged from 4.68 (BnaA06T0042800ZS) to 9.33 (BnaC03T0372400ZS). And the GRAVY was ranged from − 0.537 (BnaA09T0529500ZS) to 0.255 (BnaA03T0214100ZS). By comparing the physicochemical properties between or within groups, it was found that the physicochemical properties of BnaGH28 were similar within groups, but largely different between different groups. The average pI was greater than 7, BnaGH28 proteins in groups A, D, and E are mostly alkaline. However, proteins in groups B and C are largely acidic with the average theoretical pI < 7. The stable proteins were mainly distributed in group C. Notably, BnaGH28 proteins in groups A and D are mostly hydrophobic; while BnaGH28 proteins in groups B, C, and E are hydrophilic (Table S4).

The SL results of 37 BnaGH28 proteins have shown that there were 17 BnaGH28 proteins located in the nucleus. There were 8, 6, 4, and 2 BnaGH28 proteins located in the extracell, vacuole, chloroplast, and cytoplasm, respectively (Table S4). It was also found that the subcellular localization of BnaGH28 was mostly consistent with its corresponding homologous in *A. thaliana*. However, BnaC03T0652200ZS and BnaC03T0372400ZS, homologous to an extracellular matrix located protein (AtPGLR1 and AtPGLR2), were located on the nucleus and vacuole in present study.

### Conserved motifs and gene structure of BnaGH28

**Fig. 4:**
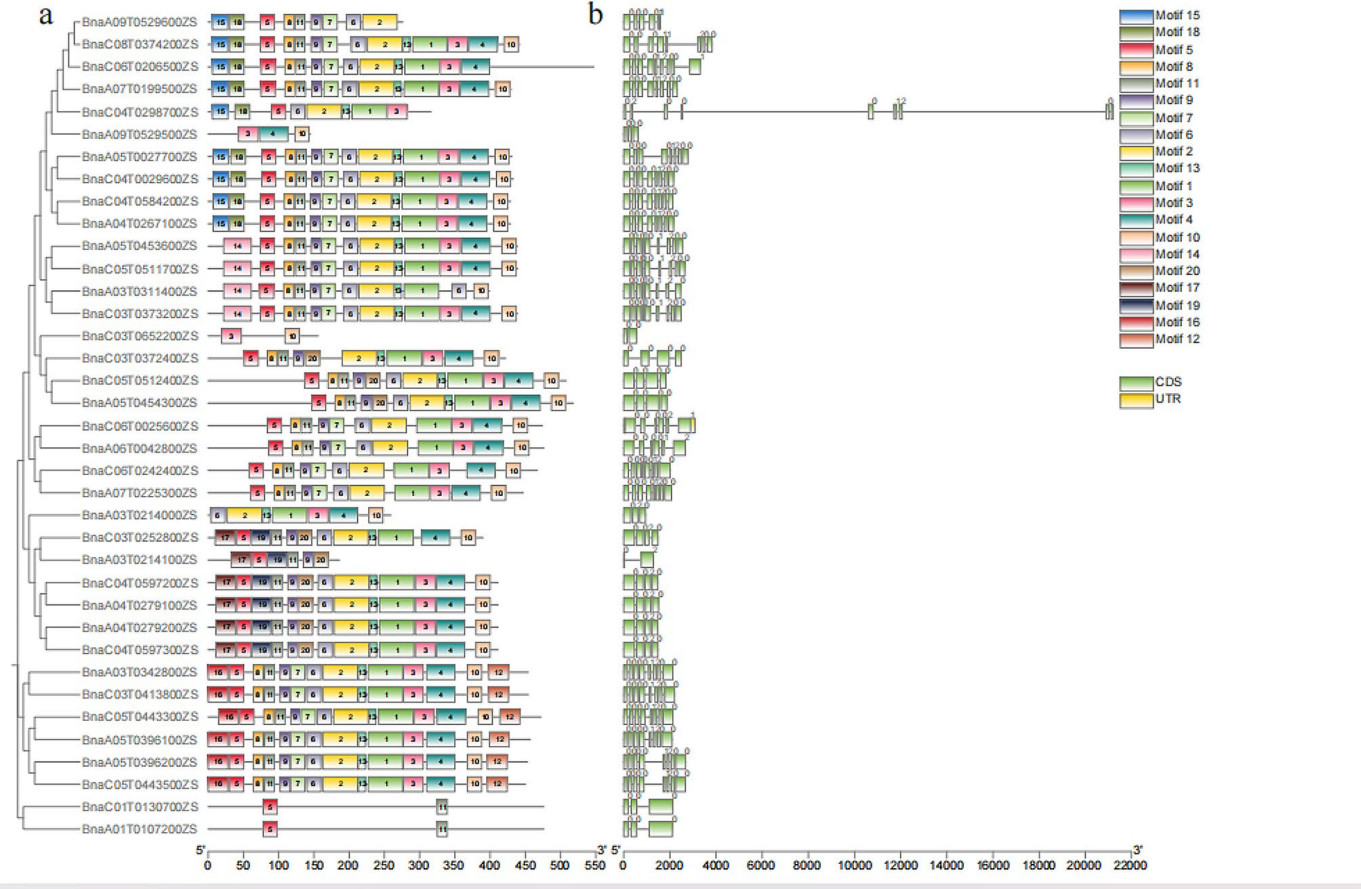
Architecture of conserved motifs and gene structures of the GH28 family in *Brassica napus* | (**a**) Conserved motifs of BnaGH28. Boxes with different colors represented different conserved motifs; (**b**) Gene structure of *BnaGH28* genes. Exons and introns are presented as filled green round-corner rectangles and thin single lines, respectively

Twenty conserved motifs of 37 BnaGH28 were obtained by using the online software MEME. We found that almost all of the BnaGH28 proteins contained motif 5 (94.59%), indicating that motif 5 is relatively conserved in the GH28 family. However, only 4 BnaGH28 proteins contained motif 14 (Fig. 4a). Three catalytic reaction related amino acid sites (CGPGHGIS, SPNTDGI, and GDDC) and ion interaction related amino acid sites (RIK) were identified in motif 1 and motif 2 (Fig. S2). And almost all 37 BnaGH28 contained these four conserved functional amino acid sites.

Results of the distribution of conserved motifs in different groups have shown that proteins in the same group have similar motif distribution. It was found that motif 17 and motif 19 mainly existed in the members of group D. BnaGH28 proteins containing motif 14, motif 15 and motif 16 were mainly in group E (Fig. 4a). These indicate that the proteins in different subgroups may have large functional differentiation.

Gene structure analysis was performed to gain a deeper understanding of the *GH28* gene expansion in *B. napus*. By aligning CDS and genomic sequences, we found that the structure and intron number were quite different among *BnaGH28* genes. It was shown that *BnaC03T0652200ZS* is the shortest one, with only 551 bp. And *BnaC04T0298700ZS* is the longest *BnaGH28*, with 21,199 bp. Most genes have 4 introns in group D, except for the *BnaA03T0214000ZS* and *BnaA03T0214100ZS* genes. The number of introns in *BnaA09T0529500ZS* was very different from other branches which have 6–9 introns in the group E (Fig. 4b).

### Tissue expression profiles of *BnaGH28* genes

**Fig. 5:**
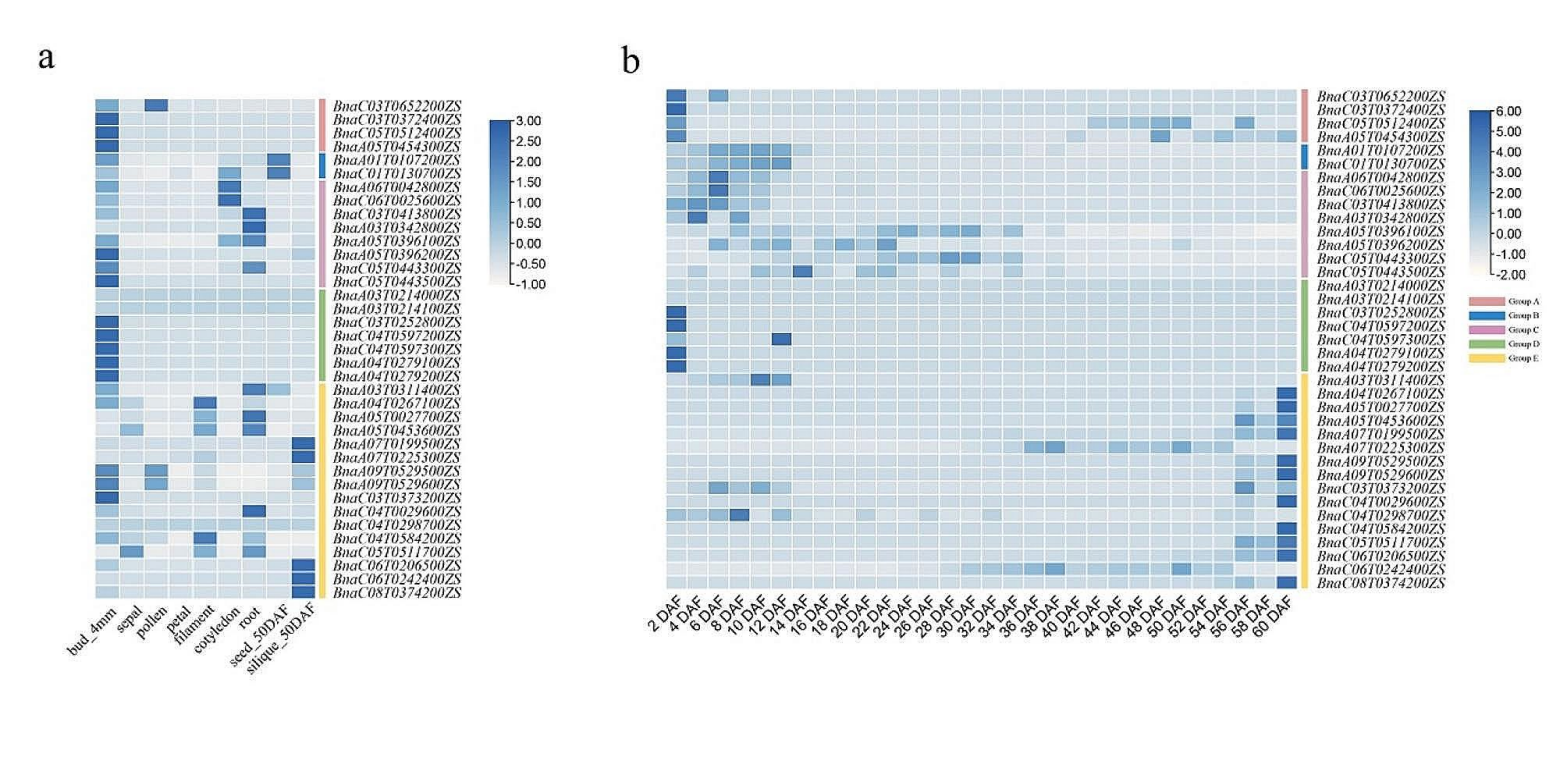
Expression profiles of *BnaGH28* genes | (**a**) Tissue expression profiles of *BnaGH28* genes; (**b**) Expression patterns of *BnaGH28* genes in developing siliques

To explore tissue-specific expression profiles of *BnaGH28* genes, the expression data of 9 tissues, including buds (4 mm), sepals, pollen, petals, filaments, cotyledons, roots, seeds (50 d after flowering) and pods (50 d after flowering), were obtained from the BnTIR database. Results have shown that only some *BnaGH28* genes in group E were highly expressed in pods (Fig. 5a). To further explore the *BnaGH28* gene expression characters during rapeseed silique development, the expression level of genes in silique at different development stages (2–60 days after flowering) were compared. Results indicated that the expression level of almost all *BnaGH28* genes in groups A, B, C, and D was decreased with the silique development. However, eleven *BnaGH28* genes in group E were highly expressed at the silique maturity stage (40–60 days after flowering) (Fig. 5b). These indicated that 11 *BnaGH28* genes might play a role in pod development of *B. napus*.

### Cis-elements and expression respond to phytohormones of *BnaGH28*

**Fig. 6:**
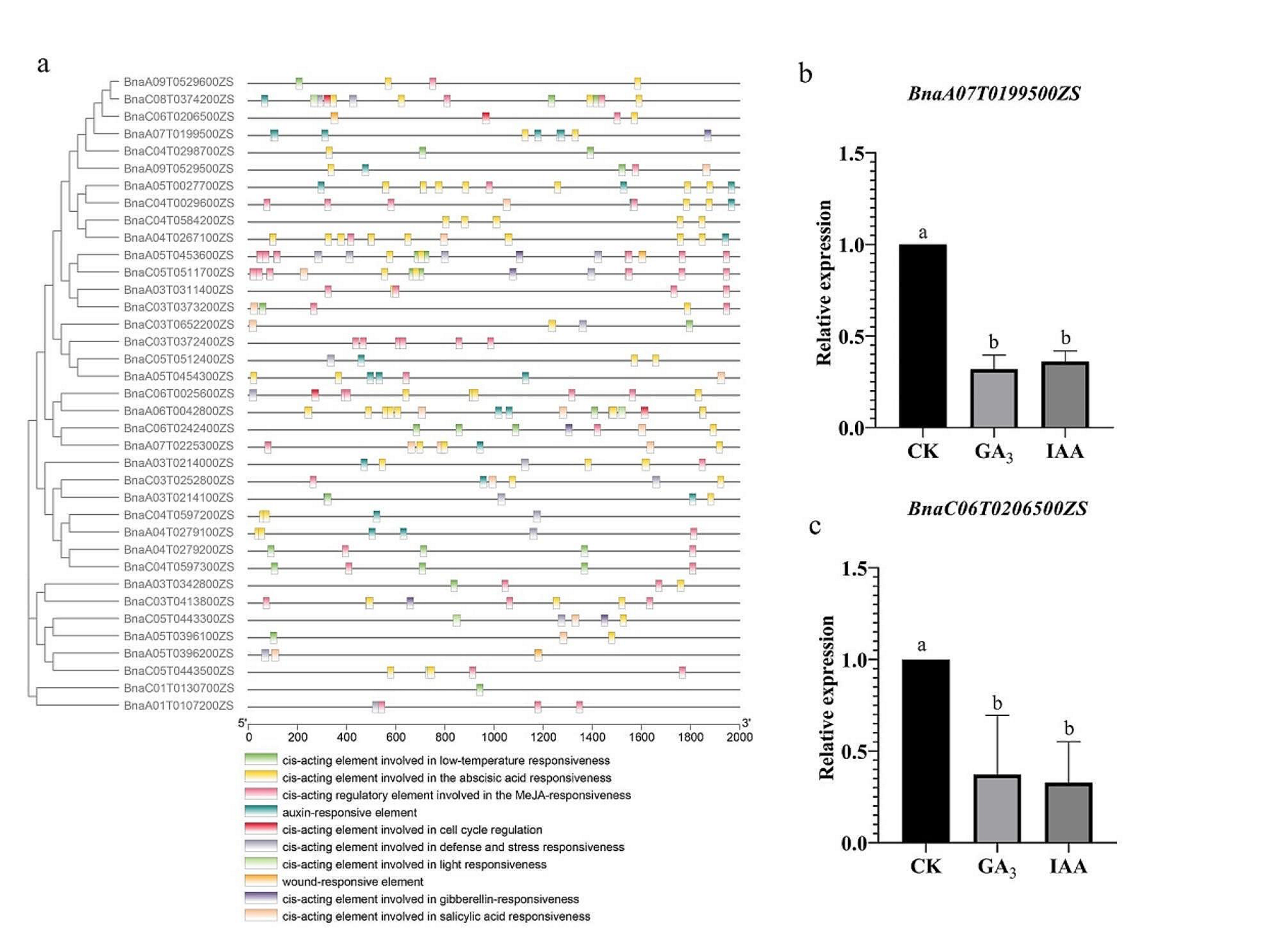
The cis-acting elements prediction of *BnaGH28* and their expression level respond to phytohormones | (**a**) The cis-acting elements distribution of *BnaGH28*; (**b**) Expression level respond to phytohormones of *BnaA07T0199500ZS*; (**c**) Expression level respond to phytohormones of *BnaC06T0206500ZS*. GA3 and IAA represent siliques treated by GA3 or IAA for 7 days, respectively

To better understand the transcriptional regulation and potential function of the *BnaGH28* genes, cis-elements in the promoter sequences were predicted by the Plant Care database. Results have demonstrated that a large number of multiple stress- or hormone-related cis-elements have been identified in the promoter region of *BnaGH28* genes (Fig. 6a). Among these, almost all *BnaGH28* genes contained hormone-related cis-acting elements (auxin response elements (TGA elements), abscisic acid responsiveness elements (ABREs), gibberellin response elements (TATC-box), salicylic acid responsiveness (TCA-element), and MeJA-responsiveness (TGACG-motif and TGTCA-motif)). There 43.75% of genes in group E have IAA response elements. Moreover, all genes in groups C or E have the ABA response elements. These indicated that the *BnGH28* expression level might be regulated by hormone signaling.

For investigating the expression level of *BnaGH28* response to phytohormones, siliques treated by GA or IAA for 7 days were employed in the present study. Only two genes’ expression levels can be detected among these 11-pod development related candidate genes (Fig. 6b-c). Results indicated that the expression levels of *BnaA07T0199500ZS* and *BnaC06T0206500ZS* were significantly downregulated by IAA and GA treatment. Thus, GA and IAA treatments might influence pod shattering resistance by regulating *BnaGH28* gene expression in *B napus*.

### Effects of *BnaGH28* variation on pod shattering resistant

**Fig. 7:**
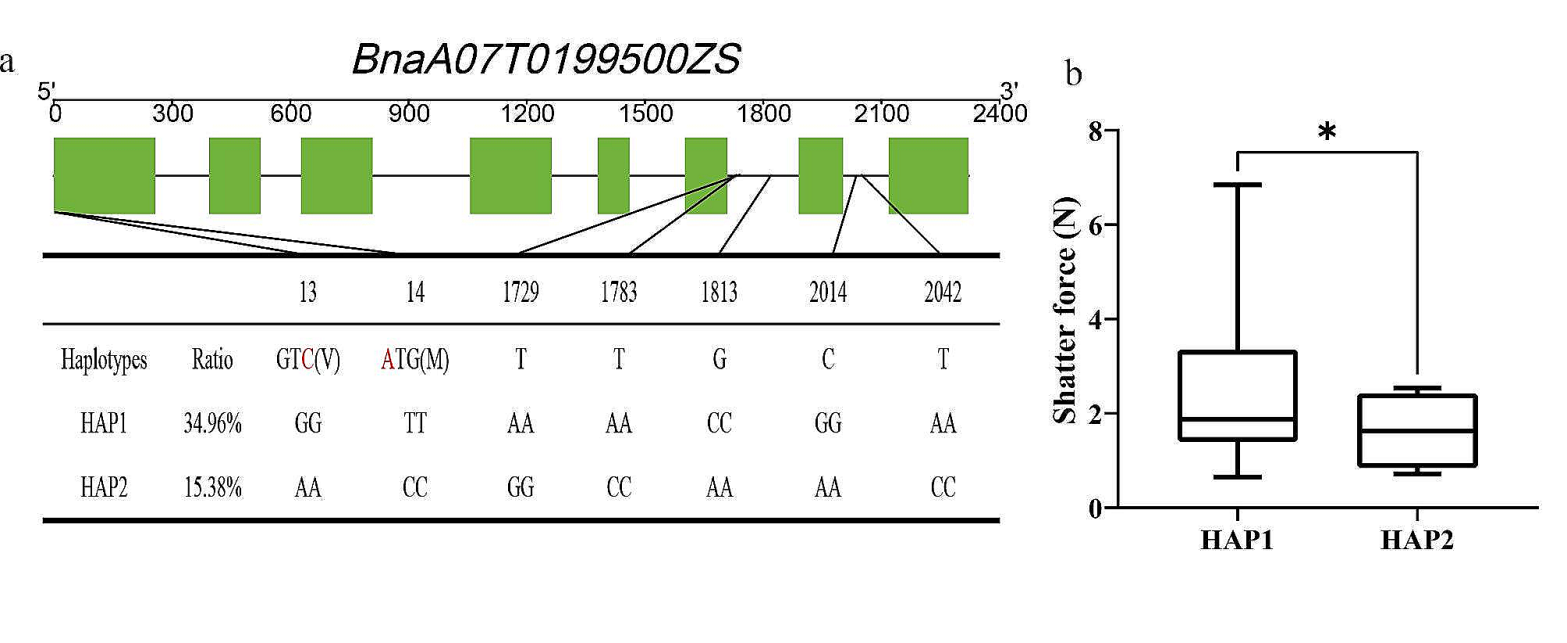
Effects of *BnaGH28* gene variation on pod shattering resistance | (**a**) Variation sites of *BnaA07T0199500ZS*; **(b**) Effects of *BnaA07T0199500ZS* variation on rapeseed pod shatter-resistance

In order to clarify the effects of *BnaGH28* gene variation on pod shattering resistance, the variation of 2 pod shattering resistance related candidate genes was investigated in a rapeseed micro-core collection. Results have shown that there were 7 variation sites in *BnaA07T0199500ZS* (Fig. 7a). However, no available variation (minor allele frequency > 0.05) was detected of *BnaC06T0206500ZS* in the present population. By comparing the pod shattering force between different variations of *BnaA07T0199500ZS*, we found that accessions carrying haplotypes 1 (Hap1) exhibited pod shattering resistance than Hap2 (Fig. 7b). These indicated *BnaA07T0199500ZS* might play vital role in pod shattering resistance in *B. napus*.

## Discussions

Pod shattering, controlled by multiple processes, is a complex and important trait in rapeseed production [29]. The *GH28* family including *PG* has been reported in pod shattering resistance in *A. thaliana* [30] and grain legumes [31]. In the current study, 37 *BnaGH28* genes were identified via genome-wide analysis. This provides a chance to clarify the role of *GH28* genes on rapeseed pod shattering resistance.

Members of the same group have a similar protein character, conserved motifs, gene structure, tissue expression profile, and cis-acting elements. These indicated that *BnaGH28s* might play an important role in pod shattering like its orthologous. *AtADPG1/2* were involved in the cell separation during *Arabidopsis* silique dehiscence; *AtQRT2* was involved in anther dehiscence in *Arabidopsis*; *AtQRT3* plays a direct role in the degradation of pollen mother cell wall [32]. However, there were also some homologous genes with different motifs and gene structures. This indicates that these genes in different subgroups may have large functional differentiation [33].

As previous study reported that promoters can regulate plant growth, development, and physiological metabolism by controlling the gene expression at the right time, place, and level [34, 35]. By analyzing the cis-elements in the *BnaGH28* family, we found that almost all *BnaGH28* genes contained hormone-related cis-acting elements in promoter region. This indicated phytohormone could regulate pod shattering resistance by affecting *BnaGH28* gene expression.

Previous report has demonstrated that IAA can influence pod shatter-resistance in *Arabidopsis* [36]. Relatively higher IAA content in cells can effectively inhibit the activity of endo-1,4-beta-glucanase and PG [37, 38]. Studies on the relationship between exogenous 2-methyl-4-chlorophenoxyacetic acid (4-CPA, an IAA analog) and pod shattering resistance have been conducted [39]. It was also be reported that the pod shattering resistance related genes *IND* and *ALC* have been influenced by GA and IAA action [14, 15, 40]. Thus, IAA or GA might play a critical role in pod shattering resistance by regulating the expression level of *BnaA07T0199500ZS* and *BnaC06T0206500ZS* in group E. Even the ABA response elements were identified in promoter region of genes in groups E. But, the effects of ABA on rapeseed pod shattering were few reported.

Furthermore, the gene allelic variations have affected pod shattering resistance in *A. thaliana* and *B. napus* [41–44]. In this study, Accessions carrying Hap1 of *BnaA07T0199500ZS* exhibited higher pod shattering resistance than that of Hap2. This indicates that the pod shattering resistance can be improved by integrating the excellent haplotypes [45]. All these findings provide critical information for investigating the molecular mechanism of *GH28* regulating pod shattering resistance in *B. napus.* And the pod shattering resistant HAP 1 of *BnaA07T0199500ZS* provide genetic resources for breeding of pod shattering resistant cultivars.

### Electronic supplementary material

Below is the link to the electronic supplementary material.

Supplementary Material 1

Supplementary Material 2

## Data Availability

The datasets used and/or analyzed during the current study are available from the corresponding author on reasonable request.
