# Supplementary material for: Genome-wide identification of GH28 family and insight into its contributions to pod shattering resistance in Brassica napus L
Source: BMC Genomics. 2024 May 17;25:492. doi: 10.1186/s12864-024-10406-y (PMC11102225; doi:10.1186/s12864-024-10406-y)
Supplement: Supplementary file 2 — Supplementary Material 2 [file 12864_2024_10406_MOESM2_ESM.docx]

**Supplementary Materials**

**Table S1: Sequences of primers used for qRT-PCR**

| Gene | Forward primer | Reverse primer |
| --- | --- | --- |
| BnaA04T0267100ZS | TTCACTTCTCTACGTACCAACC | TTTCTTGAAGCTTCACACCATG |
| BnaA05T0027700ZS | CGCAACAGATTCAGATTTCGAT | TGTGTATACCATCGGTGTTAGG |
| BnaA05T0453600ZS | GGTGGCCAAATTCTTGCAAGAT | TGGGGACATGATTCAACACGAT |
| BnaA07T0199500ZS | GAAAAACCGATGATACTCAGGC | GGATCTGGAAGCTACGTAATGA |
| BnaA09T0529500ZS | GGGGATGACAATTCCAAAGCTT | TGAGGAGAAACGTTGCCTTGAT |
| BnaA09T0529600ZS | TTATATTTTGACTCAGCGGTGC | CACTGCAATCAAACGTTATTGC |
| BnaC04T0029600ZS | AACAGTCTGTCAGTAGATGGTG | ATCGAAATCTGAATCTGTTGCG |
| BnaC04T0584200ZS | ATGGCTCATTGTTGCACAGTTC | CTTCGTTCCGTTTGCATGAGTT |
| BnaC05T0511700ZS | GTCGTGGTTCCGAAAAATAGAG | CTTTCCATGCCTCAATCTTTCC |
| BnaC06T0206500ZS | AATCTATCAATCGATGGCGGCT | TGTTCTTAGCAGTTCCTGACCC |
| BnaC08T0374200ZS | AAATGCGAACAACAAGACTCTG | CACTGCAATCAAACGTTATTGC |
| Actin | AACCTTCTCTCAAGTCTCTGTG | CCAGAATCATCACAAAGCATCC |

**Table S3: The conversed domain of GH28 in *A. thaliana* and *B. napus***

| Gene Name | Domain of GH28 genes | | |
| --- | --- | --- | --- |
| QRT2 | GH28 | [PbH1]_4_ | — |
| ADPG2 | GH28 | [PbH1]_5_ | Pectate lyase 3 |
| ADPG1 | GH28 | [PbH1]_5_ | Pectate lyase 3 |
| QRT3 | — | [PbH1]_3_ | Pectate lyase 3 |
| PGLR4 | GH28 | [PbH1]_5_ | — |
| PGLR1 | GH28 | [PbH1]_5_ | Pectate lyase 3 |
| PGLR2 | GH28 | [PbH1]_5_ | Pectate lyase 3 |
| PGLR3 | GH28 | [PbH1]_6_ | Pectate lyase 3 |
| PGLR5 | GH28 | [PbH1]_6_ | — |
| PGLR6 | GH28 | [PbH1]_3_ | Pectate lyase 3 |
| BnaA05T0453600ZS | GH28 | [PbH1]_4_ | — |
| BnaA03T0311400ZS | [GH28]_2_ | [PbH1]_3_ | [Pectate lyase 3]_3_ |
| BnaC05T0511700ZS | GH28 | [PbH1]_4_ | — |
| BnaC03T0373200ZS | GH28 | [PbH1]_4_ | — |
| BnaA05T0027700ZS | GH28 | [PbH1]_5_ | [Pectate lyase 3]_2_ |
| BnaC04T0029600ZS | [GH28]_2_ | [PbH1]_5_ | [Pectate lyase 3]_2_ |
| BnaC04T0584200ZS | GH28 | [PbH1]_6_ | [Pectate lyase 3]_2_ |
| BnaA04T0267100ZS | GH28 | [PbH1]_5_ | [Pectate lyase 3]_2_ |
| BnaA09T0529500ZS | GH28 | [PbH1]_2_ | — |
| BnaC06T0206500ZS | [GH28]_2_ | [PbH1]_5_ | [Pectate lyase 3]_2_ |
| BnaA09T0529600ZS | GH28 | PbH1 | Pectate lyase 3 |
| BnaC08T0374200ZS | GH28 | [PbH1]_5_ | [Pectate lyase 3]_2_ |
| BnaA07T0199500ZS | [GH28]_2_ | [PbH1]_6_ | [Pectate lyase 3]_2_ |
| BnaC04T0298700ZS | GH28 | [PbH1]_5_ | [Pectate lyase 3]_2_ |
| BnaC01T0130700ZS | — | [PbH1]_3_ | Pectate lyase 3 |
| BnaA01T0107200ZS | — | [PbH1]_3_ | [Pectate lyase 3]_2_ |
| BnaC06T0025600ZS | GH28 | [PbH1]_5_ | — |
| BnaA06T0042800ZS | GH28 | [PbH1]_5_ | — |
| BnaC03T0652200ZS | GH28 | — | — |
| BnaC03T0372400ZS | GH28 | [PbH1]_6_ | [Pectate lyase 3]_2_ |
| BnaC05T0512400ZS | GH28 | [PbH1]_5_ | Pectate lyase 3 |
| BnaA05T0454300ZS | GH28 | [PbH1]_5_ | Pectate lyase 3 |
| BnaC05T0443300ZS | GH28 | [PbH1]_5_ | [Pectate lyase 3]_2_ |
| BnaA05T0396200ZS | GH28 | [PbH1]_5_ | [Pectate lyase 3]_2_ |
| BnaC05T0443500ZS | GH28 | [PbH1]_5_ | Pectate lyase 3 |
| BnaA03T0342800ZS | GH28 | [PbH1]_5_ | Pectate lyase 3 |
| BnaA05T0396100ZS | GH28 | [PbH1]_5_ | [Pectate lyase 3]_2_ |
| BnaC03T0413800ZS | GH28 | [PbH1]_5_ | Pectate lyase 3 |
| BnaC06T0242400ZS | [GH28]_2_ | [PbH1]_5_ | [Pectate lyase 3]_3_ |
| BnaA07T0225300ZS | GH28 | [PbH1]_5_ | [Pectate lyase 3]_3_ |
| BnaA03T0214000ZS | GH28 | [PbH1]_4_ | — |
| BnaC04T0597200ZS | GH28 | [PbH1]_4_ | — |
| BnaA04T0279200ZS | GH28 | [PbH1]_5_ | [Pectate lyase 3]_2_ |
| BnaC04T0597300ZS | GH28 | [PbH1]4 | Pectate lyase 3 |
| BnaC03T0252800ZS | [GH28]_2_ | [PbH1]4 | Pectate lyase 3 |
| BnaA04T0279100ZS | GH28 | [PbH1]5 | [Pectate lyase 3]_2_ |
| BnaA03T0214100ZS | GH28 | — | [Pectate lyase 3]_2_ |

Note：GH28: Glycoside hydrolase 28 domain (PF00295); PbH1: Parallel beta-helix repeats domain (SM00710); Pectate lyase 3: Pectinate lyase 3 domains (PF12708); [X]n: It indicates that there are n X domains in the GH28 gene.；-: It indicates that this domain does not exist in the GH28 gene.

**Table S4: Physicochemical properties of BnaGH28 proteins**

| Group | Proteins | AA | MW (kD) | pI | II | GRAVY | SL |
| --- | --- | --- | --- | --- | --- | --- | --- |
| Group A | BnaC01T0130700ZS | 475 | 51043.93 | 5.77 | 17.67 | 0.015 | extr |
|  | BnaA01T0107200ZS | 475 | 51088.91 | 5.52 | 19.48 | 0.022 | cyto |
| Group B | BnaC05T0443300ZS | 471 | 50638.25 | 5.53 | 36.23 | -0.063 | nucl |
|  | BnaA05T0396200ZS | 452 | 48441.71 | 5.99 | 31.21 | -0.097 | extr |
|  | BnaC05T0443500ZS | 449 | 48052.31 | 6.08 | 28.91 | -0.117 | extr |
|  | BnaA03T0342800ZS | 453 | 48521.73 | 5.42 | 35.58 | -0.075 | vacu |
|  | BnaA05T0396100ZS | 456 | 48663.02 | 5.16 | 35.13 | -0.044 | extr |
|  | BnaC03T0413800ZS | 453 | 48514.72 | 5.33 | 35.15 | -0.053 | chlo |
| Group C | BnaC06T0025600ZS | 473 | 50893.19 | 4.74 | 49.1 | -0.19 | nucl |
|  | BnaA06T0042800ZS | 475 | 51217.53 | 4.68 | 50.59 | -0.213 | nucl |
| Group D | BnaC03T0652200ZS | 155 | 16720.28 | 9.15 | 22.52 | -0.023 | nucl |
|  | BnaC03T0372400ZS | 421 | 45093.23 | 9.33 | 28.59 | -0.3 | vacu |
|  | BnaC05T0512400ZS | 507 | 52923.64 | 8.98 | 24.44 | -0.248 | extr |
|  | BnaA05T0454300ZS | 517 | 54140.16 | 9.07 | 23.8 | -0.281 | extr |
|  | BnaA03T0214000ZS | 258 | 27645.29 | 6.22 | 37.71 | -0.078 | nucl |
|  | BnaC04T0597200ZS | 410 | 44109.81 | 8.92 | 32.45 | 0.03 | vacu |
|  | BnaA04T0279200ZS | 410 | 43872.22 | 7.04 | 34.57 | 0.006 | extr |
|  | BnaC04T0597300ZS | 410 | 43922.33 | 7.49 | 33.31 | 0.025 | vacu |
|  | BnaC03T0252800ZS | 389 | 41268.93 | 6.16 | 30.57 | 0.083 | extr |
|  | BnaA04T0279100ZS | 410 | 44314.06 | 8.89 | 33.76 | 0.014 | vacu |
|  | BnaA03T0214100ZS | 185 | 20020.3 | 9.05 | 24.33 | 0.255 | cyto |
| Group E | BnaA05T0453600ZS | 438 | 47816.53 | 9.21 | 40.64 | -0.253 | nucl |
|  | BnaA03T0311400ZS | 398 | 43835.01 | 9.27 | 36.91 | -0.283 | nucl |
|  | BnaC05T0511700ZS | 438 | 47770.23 | 9 | 39.88 | -0.268 | nucl |
|  | BnaC03T0373200ZS | 438 | 48468.11 | 8.99 | 38.36 | -0.347 | nucl |
|  | BnaA05T0027700ZS | 430 | 46337.12 | 8.6 | 29.97 | -0.29 | nucl |
|  | BnaC04T0029600ZS | 432 | 46578.4 | 8.68 | 30.94 | -0.291 | nucl |
|  | BnaC04T0584200ZS | 428 | 46003.85 | 8.67 | 29.4 | -0.299 | nucl |
|  | BnaA04T0267100ZS | 428 | 46052.79 | 8.5 | 29.71 | -0.305 | nucl |
|  | BnaA09T0529500ZS | 144 | 15501.33 | 8.18 | 30.5 | -0.537 | chlo |
|  | BnaC06T0206500ZS | 546 | 58893.73 | 8.79 | 35.4 | -0.271 | nucl |
|  | BnaA09T0529600ZS | 275 | 30095.08 | 8.54 | 31.73 | -0.362 | vacu |
|  | BnaC08T0374200ZS | 441 | 48040.14 | 8.22 | 29.21 | -0.43 | nucl |
|  | BnaA07T0199500ZS | 429 | 46347.23 | 8.23 | 25.19 | -0.374 | nucl |
|  | BnaC04T0298700ZS | 315 | 34158.83 | 4.97 | 29.24 | -0.438 | nucl |
|  | BnaC06T0242400ZS | 466 | 50779.55 | 8.78 | 42.9 | -0.19 | chlo |
|  | BnaA07T0225300ZS | 446 | 48645.2 | 8.77 | 41.09 | -0.159 | chlo |

Note: AA: Number of amino acids; pI: Isoelectric point; MW: Molecular weight; II: Instability index; GRAVY: Grand average of hydropathicity; SL: Subcellular Localization. nucl: nucleus; chlo: chloroplast; extr: extracell; vacu: vacuole; cyto: cytoplasm.

**
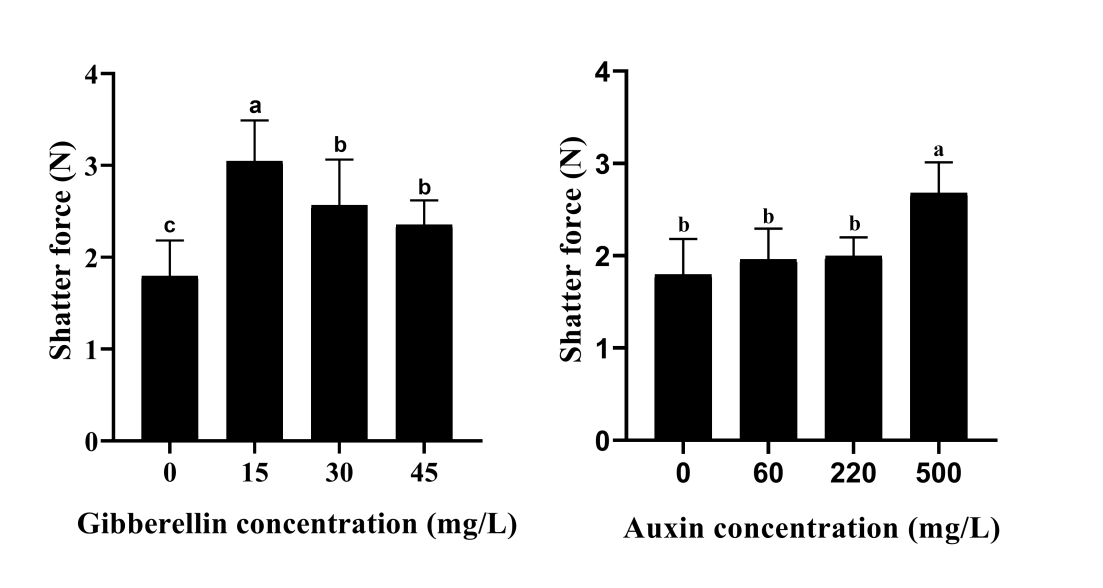
**

**Figure S1. The effect of GA and IAA on pod shattering resistance**


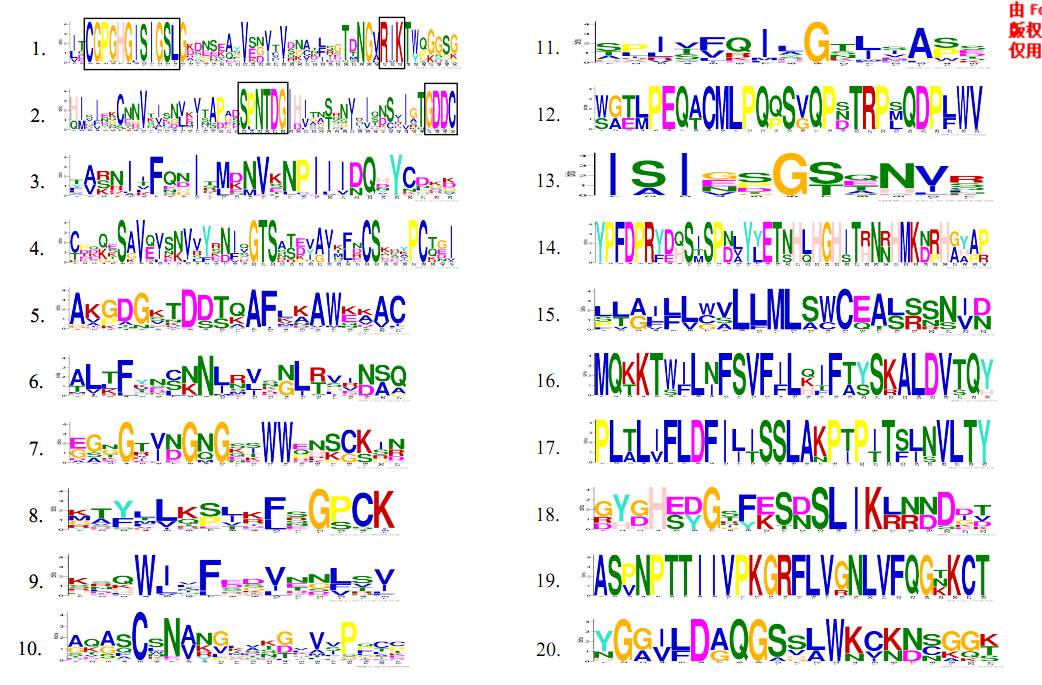


**Figure S2. Conserved motifs in *BnaGH28s***
